# TNFa and IL2 Encoding Oncolytic Adenovirus Activates Pathogen and Danger-Associated Immunological Signaling

**DOI:** 10.3390/cells9040798

**Published:** 2020-03-26

**Authors:** Camilla Heiniö, Riikka Havunen, Joao Santos, Klaas de Lint, Victor Cervera-Carrascon, Anna Kanerva, Akseli Hemminki

**Affiliations:** 1 Cancer Gene Therapy Group, Faculty of Medicine, TRIMM, University of Helsinki, Haartmaninkatu 3, 00290 Helsinki, Finland; camilla.heinio@helsinki.fi (C.H.); joao.santos@helsinki.fi (J.S.); victor.cerveracarrascon@helsinki.fi (V.C.-C.); anna.kanerva@helsinki.fi (A.K.); 2 TILT Biotherapeutics Ltd., Haartmaninkatu 3, 00290 Helsinki, Finland; riikka.havunen@helsinki.fi; 3 Cancer Center Amsterdam, Department of Clinical Genetics, Section Oncogenetics, Amsterdam UMC, De Boelelaan 1117, 1118, 1081 HV Amsterdam, The Netherlands; k.delint@amsterdamumc.nl; 4 Department of Obstetrics and Gynecology, Helsinki University Hospital, Haartmaninkatu 2, 00290 Helsinki, Finland; 5 Helsinki University Hospital Comprehensive Cancer Center, Paciuksenkatu 3, 00290 Helsinki, Finland

**Keywords:** aenovirus, virotherapy, immunotherapy, AIM2, oncolytic virus, TILT-123, immunological cell death, DAMP, PAMP

## Abstract

In order to break tumor resistance towards traditional treatments, we investigate the response of tumor and immune cells to a novel, cytokine-armed oncolytic adenovirus: Ad5/3-d24-E2F-hTNFa-IRES-hIL2 (also known as TILT-123 and OAd.TNFa-IL2). There are several pattern recognition receptors (PRR) that might mediate adenovirus-infection recognition. However, the role and specific effects of each PRR on the tumor microenvironment and treatment outcome remain unclear. Hence, the aim of this study was to investigate the effects of OAd.TNFa-IL2 infection on PRR-mediated danger- and pathogen-associated molecular pattern (DAMP and PAMP, respectively) signaling. In addition, we wanted to see which PRRs mediate an antitumor response and are therefore relevant for optimizing this virotherapy. We determined that OAd.TNFa-IL2 induced DAMP and PAMP release and consequent tumor microenvironment modulation. We show that the AIM2 inflammasome is activated during OAd.TNFa-IL2 virotherapy, thus creating an immunostimulatory antitumor microenvironment.

## 1. Introduction

By selective replication in tumor cells, oncolytic viruses create an immunostimulatory tumor microenvironment, which has been widely documented to be essential for successful cancer immunotherapy [1,2]. Besides tumor-bursting lytic capabilities, oncolytic viruses have additional, intrinsic immune-activating properties, thus utilizing two separate mechanisms to stop tumor growth. Furthermore, most oncolytic viruses can relatively easily be modified to augment their intrinsic antitumoral effects [3]. For example, Amgen’s herpes virus Talimogene Laherparepvec (Imlygic), which is modified to encode Granulocyte Macrophage Colony-Stimulating Factor (GM-CSF), has shown good efficacy melanoma, and it has been approved for clinical use in the USA and the EU [4,5].

Similarly, the immunostimulating properties of the adenovirus studied here have been enhanced by the addition of two cytokine coding genes: IL-2 and TNFa. Consequently, when expressed, these two cytokines increase T-cell infiltration and stimulation in a tumor [6], both of which have been associated with positive patient outcomes [7,8]. However, the exact molecular mechanism behind the noted antitumor efficacy of virotherapy and OAd.TNFa-IL2 virus specifically is still poorly understood. Therefore, in order to shed light on this issue, the molecular mechanisms causing antitumoral effects were mapped in this study.

Several receptors and sensors have been identified as in-cell alarm systems, which inform the cell of foreign entities, like viruses, capsid proteins, or extranuclear DNA. These receptors are called pattern recognition receptors (PRR). The engagement of these receptors creates a signal that activates the innate immune system, enhancing antigen processing and stimulating the adaptive immune system. Simultaneously, these signals prime and enhance antitumoral immunity. Therefore, PRR-receptors such as Toll-like receptors (TLR), nucleotide-binding oligomerization domain-like receptors (NOD-like receptors), the stimulator of interferon genes (STING) pathway and inflammasomes, such as absent in melanoma 2 (AIM2), have been shown to play an important role in the intratumoral signaling and tumor growth inhibition and might thus have a marked effect on patient outcome [9,10,11]. For example, TLR9 recognizes intracellular cytosomal non-methylated DNA, such as adenovirus DNA. The activation of this receptor’s downstream signaling cascade leads to the activation of NFκB signaling and eventual cytokine release (such as TNFa, IL-6, and IL-12). The effects of NFκB-signaling on patient outcome are yet to be determined, but some studies indicate that chronic activation promotes a tumor growth-favorable environment. For example, studies have shown that NFκB activity can promote tumor cell, can suppress apoptosis, and can enhance vascular growth [12,13]. Furthermore, it can energize the immune system in certain circumstances [13]. However, contradicting results for its role in cancer has also been documented; one recurring theme in immunology is that chronic proinflammatory signaling can contribute to carcinogenesis, while acute signaling can cause antitumor immune responses [14].

Inflammasomes, such as AIM2, are proteins that, upon stimulation, multimerize and react to intracellular oddities, such as cytosolic DNA. Binding of dsDNA to the HIN domain of AIM2 releases the amino-terminal pyrin domain (PYD), leading to the recruitment and multimerization of apoptosis-associated speck-like protein containing a caspase recruitment domain (ASC) proteins and to the recruitment and activation of caspase-1, which in turn modifies gasdermin and IL-1beta to their active forms [15,16]. De Konig et al. showed a tumor-suppressive role for AIM2 when they studied both epithelial and melanocytic lesions [17]. Furthermore, a lack of AIM2 expression has been suggested to cause poor patient outcome in colorectal cancer [11]. Additionally, AIM2 activation has an NFκB-inhibiting and consequently cell growth-impeding function. However, thorough research still needs to be done on the role of AIM2 in order to avoid erroneous generalized conclusions.

Here, we studied danger- and pathogen-associated molecular pattern (DAMP and PAMP, respectively) signaling in the context of OAd.TNFa-IL2 virotherapy. We screened receptors and signaling cascades activated upon OAd.TNFa-IL2 infection, which could be responsible for OAd.TNFa-IL2 -induced antitumor efficacy. The results give insight into the biology and clinical use of adenoviruses, TNFa, and IL-2.

## 2. Results

### 2.1. OAd.TNFa-IL2 Induces Immunostimulating Microenvironmental Changes Through DAMP and PAMP Release and Expression

**Figure 1:**
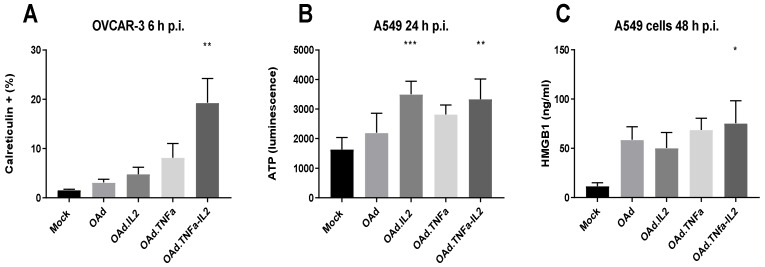
OAd.TNFa-IL2 induces alarmin release associated with danger- and pathogen-associated molecular pattern (DAMP and PAMP, respectively) signaling. Different cell lines were infected with an unarmed oncolytic adenovirus (OAd), Ad5/3-E2F-d24-hIL2 (OAd.IL2), Ad5/3-E2F-d24-hTNFa (OAd.TNFa), or Ad5/3-E2F-d24-hTNFa-IRES-hIL2 (OAd.TNFa-IL2) 100 VP/cell. The release of DAMP and PAMP molecules (**A**) calreticulin was measured by flow cytometry, and (**B**) ATP and (**C**) HMGB1 were measured by ELISA. The data are presented as mean + SEM; the group size was *n* > 4. Statistical significance was determined by Kruskal–Wallis test; groups were compared to mock. * *p* < 0.05; ** *p* < 0.01; *** *p* < 0.001.

To determine if OAd.TNFa-IL2 virotherapy affects the innate immunity and the microenvironment, we measured three established DAMP- and PAMP-reporter molecules also called alarmins: calreticulin, HMGB1, and extracellular ATP. We used three cell lines representing different cancer types: lung adenocarcinoma A549, ovarian cancer OVCAR-3, and melanoma SK-MEL-28. Early, medium, and late time points (6 h, 24 h, and 48 h postinfection) were selected to determine both immediate and long-term effects of the infection. OAd.TNFa-IL2 induced alarmin release at several time points (Supplementary Figure S1). The infection induced significant cell surface expression of calreticulin at 6 h compared to mock treated cells *p* = 0.0032 (Figure 1A). Extracellular ATP levels were raised 24 h after infection (OAd.TNFa-IL2 vs. mock *p* = 0.002) (Figure 1B) and extracellular HMGB1 levels were raised after 48 h (OAd.TNFa-IL2 vs. mock *p* = 0.0114). These results indicate that OAd.TNFa-IL2 might create an immunostimulating signal through DAMP and PAMP release in the tumor.

### 2.2. Oncolytic Adenovirus Therapy Affects the Transcriptome Landscape of Both Cancer and Immune Cells

Since alarmin release and the presence of OAd.TNFa-IL2 can activate several different receptors and downstream signaling molecules, mRNA sequencing was employed to pinpoint which DAMP- and PAMP-signaling cascades in both cancer cells (SK-MEL-28 melanoma cells) and dendritic cells (DCs) are reactive to OAd.TNFa-IL2 virotherapy. Adenovirus infection caused an upregulation of several toll-like receptor signaling cascades: TLR1, 2, 3, 5, 7, and 9. Additionally, inflammasome receptor AIM2 was upregulated in unfiltered RNA sequencing results in both SK-MEL-28 and DC (Supplementary Table S1). However, once the results were filtered for the possible bias of the sequencing system, only a few DAMP- and PAMP-signaling cascade genes were upregulated. Of note, AIM2 was upregulated in SK-MEL28 also after filtering (Supplementary Table S1). Additionally, cytokines IL-1beta and IL-18, which are released after AIM2 activation, were also highly upregulated compared to mock. Therefore, we further investigated the role of AIM2 during our virotherapy regime.

Table 1 presents the most upregulated genes during OAd.TNFa-IL2 adenovirus infection. Interestingly, OAd.TNFa-IL2 -encoded cytokines IL-2 and TNFa were the two most expressed genes in SK-MEL-28 cells during infection. This indicates active virus replication and multiplication, plausibly ending in a cancer cell lysis. There was an over 11-fold increase in the expression levels of IL-2 and TNFa during infection as compared with noninfected SK-MEL-28 cells. Regarding DCs, which are not malignant cells, it has been recorded that, while 5/3 chimeric, d24 adenoviruses can be internalized by these cells, this does not lead to productive replication, since the virus is tumor selective [18]. This notion is supported by our results, as IL-2 and TNFa mRNA levels in dendritic cells were up 1.6-fold for IL-2 while the TNFa expression was not upregulated significantly. This implies that the replication of OAd.TNFa-IL2 is cancer-cell specific as designed and does not harm normal cells such as DCs.

The third most affected gene in SK-MEL-28 cells was ubiquitin, which has a role in protein degradation and successful adenovirus replication. Ubiquitin showed over 9-fold higher expression in infected cells compared to mock-treated cells.

The genes that were subjected to the highest gene expression level changes in DCs were less well known. Myotrophin was the most upregulated gene in infected DCs with almost 10-fold increase in expression. Although the effect of myotrophin in DCs is not well studied, the expression seems to have an inhibitory effect on NFκB signaling, which in turn has in some cancer studies been seen as a beneficial trait [19]. Moreover, the gene expression level of CXCL9, a potent leukocyte chemotaxis molecule, was up threefold in DCs. Overall, these gene expression changes indicate that OAd.TNFa-IL2 induces favorable changes in the tumor by affecting both the innate response through the infected cells and the adaptive immune system.

### 2.3. NFκB-Related Genes Are Downregulated During OAd.TNFa-IL2 Infection, But TLR9 Signaling Is Not Activated by OAd.TNFa-IL2

To study the correlation of mRNA and functional protein expression, we analyzed protein levels in infected SK-MEL-28 cells by ELISA, NFκB pathway protein analysis, and HEK-Blue-hTLR9 cell analysis.

**Figure 2:**
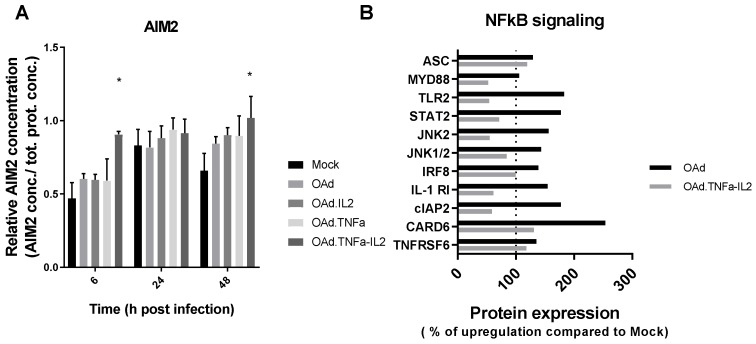
Protein expression measurements: (**A**) The AIM2 protein levels measured from infected (100 VP/cell) SK-MEL-28 cells with indicated viruses at several timepoints by ELISA. Kruskal–Wallis SEM compared to mock, * *p* = 0.05. (**B**) Infected SK-MEL28 cells were analyzed for NFκB-related protein expression at 48 h post infection.(p.i.) (100 VP/cell). Kruskal–Wallis SEM compared to mock, * *p* = 0.05.

ELISA results showed a higher relative expression of AIM2 6 and 48 h postinfection (Figure 2A). Additionally, the AIM2 protein ASC was upregulated to 119.5% compared to mock cell cultures, while the NFκB proteins were mostly downregulated during infection (Figure 2B). Interestingly, the activation of AIM2 is enhanced by arming OAd with both TNFa and IL2, even though this trend is not statistically significant.

**Figure 3:**
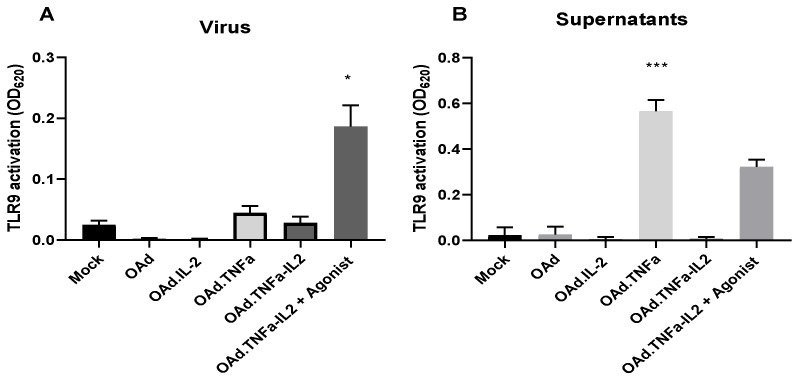
Assessment of virotherapy-induced TLR9 activation in HEK-blue TLR9 cells: HEK-blue hTLR9 cells were treated with either viruses or supernatants collected from infected cell cultures for 8 h and analyzed spectrophotometrically. Agonist = ODN2006 (synthetic, unmethylated CpG dinucleotides). The data are shown as means + SEM, *n* ≥ 4. Statistical significance is determined by Kruskal–Wallis test, treatment compared with mock * *p* < 0.05; *** *p* < 0.001.

HEK-blue hTLR9 cells were employed to analyse if virotherapy induced TLR9 activation. OAd.TNFa-IL2 or cell culture supernatant collected from infected cells did not significantly induce TLR9 pathway activation, as evaluated with indicator HEK-blue cells (Figure 3). Additionally, the effect of single armed viruses on TLR9 activation was investigated. Only TNFa supernatants were shown to activate the TLR9 signaling cascade, plausibly through the supernatant containing the lysis products (including TNFa) of cells lysed by the viruses. These conclusions were supported by the RNA-seq results (Supplementary Table S1), indicating that TLRs are not in a main role in recognizing OAd.TNFa-IL2 infection and in facilitating microenvironmental changes. All in all, the results show that the affected cascades are mostly NFκB signaling independent and the signal is conveyed through receptors other than TLR9.

### 2.4. Knockout of AIM2 Diminishes the Effect of Virotherapy

In order to determine the role of AIM2 in antitumor efficacy and its effect on tumor cytokine composition, we implanted either AIM2−/− knockout (KO) or wild-type (wt) HapT1 tumors in Syrian hamsters’ flanks. The knockout cell line was CRISPR/Cas9 created, and the deletion of AIM2 was confirmed by sequencing (Supplementary Figure S5).

**Figure 4:**
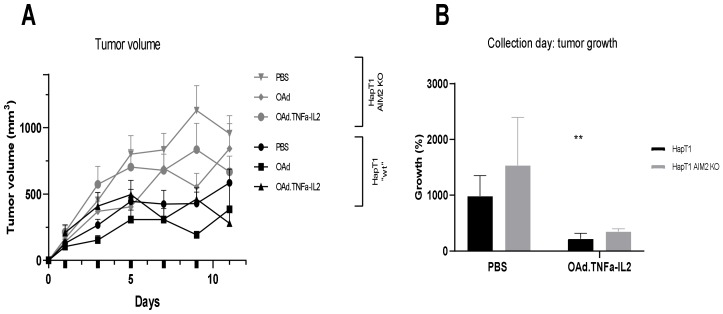
In vivo assessment of AIM2 knockout (KO) effect on tumor growth: Syrian hamster subcutaneous HapT1 or HapT1 AIM2−/− tumors were treated every other day (treatment days indicated as bold ticks on x-axis) with indicated viruses or control until collection of samples on day 11 of the experiment. (**A**) Tumor growth measured in volume. (**B**) Percentual growth of tumors on experiment day 11 compared to the size on experiment day 1: Statistical analysis was conducted compared to PBS treated, “wt” HapT1 tumors by Kruskal–Wallis test; mean + SEM are shown, ** *p* < 0.01.

Subcutaneous tumors were treated with virotherapy five times, with OAd.TNFa-IL2 or unarmed virus or mock treatment. The KO tumors showed a trend of more aggressive tumor growth. Mock KO tumors grew bigger than wild-type mock tumors, having a mean volume of 533 mm^3^ compared to KO tumors with a mean volume of 956 mm^3^ (*p* = 0.0397). Overall, OAd.TNFa-IL2 hindered tumor growth in both tumor types (Figure 4).

### 2.5. Virotherapy Induces AIM2-Dependent, Pro-Inflammatory Tumor Landscape Modification In Vivo

**Figure 5:**
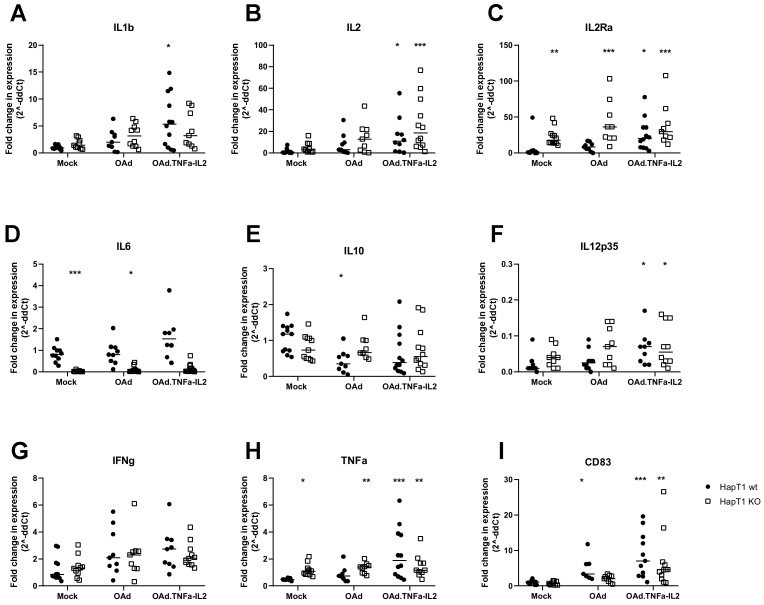
Determination of tumor microenvironment cytokine gene expression changes due to virotherapy: Virotherapy-treated subcutaneous HapT1 (AIM2 −/− KO or “wt”) tumors were collected and analyzed by RTqPCR for indicated markers of the immunological status of the tumor. (**A**) IL1b, (**B**) IL2, (**C**) IL2Ra, (**D**) IL6, (**E**) IL10, (**F**) IL12p35, (**G**) INFg, (**H**) TNFa, and (**I**) CD83. Statistical significance towards the wt mock control was analyzed by Kruskal–Wallis test * *p* < 0.05; ** *p* < 0.01, *** *p* < 0.001, after ROUT outlier (Q = 1%) removal; lines represent median values.

HAPT1 or HAPT1 AIM2 KO tumors were collected and analyzed by reverse transcriptase (RT) qPCR (Figure 5). Importantly, IL-1beta was upregulated in wt OAd.TNFa-IL2-treated group, indicating activation of AIM2 signaling cascade (Figure 5A), which was diminished by KO. IL-2 concentrations were higher in both cell types treated with the OAd.TNFa-IL2 probably due to virus replication and virus-induced signaling (Figure 5B), Also, KO of AIM2 and both virotherapies increased intraturmoral IL2Ra levels (Figure 5C). IL-10 levels remained the same between all groups, except for OAd treated wt cells, where it significantly lowered the expression (Figure 5D). The cytokine IL-6 was among the most affected by AIM2 KO. Its expression was significantly lower in HAPT1 KO tumors (Figure 5E). OAd.TNFa-IL2 infection caused significantly higher IL-12p35 levels in both wt and KO cells compared to mock treated wt tumors (Figure 5F). As expected, virus treatments caused a trend of higher IFNg expression in the virotherapy treated tumors (Figure 5G). TNFa, expression was upregulated in all KO groups and in wt OAd.TNFa-IL2-treated tumors. Interestingly, the DC maturation marker CD83 was significantly more expressed in wt OAd and OAd.TNFa-IL2-treated tumors as well as KO OAd.TNFa.IL2-treated tumors, indicating that virus-induced AIM2-mediated tumor-cell signaling might play a role in DC activation. The noted higher values could either be caused by an influx of DCs due to the virus or alternatively due to maturation of the already infiltrated DCs upon infection.

## 3. Materials and Methods

### 3.1. Cell Lines and Viruses

A549, OVCAR-3, and SK-MEL-28 cell lines were acquired from the ATCC cell bank, while hamster pancreatic cancer HapT1 was acquired from DSMZ. Cells were cultured in recommended conditions: Roswell Park Memorial Institute medium (RPMI) 1640 or Dulbecco’s modified Eagles’s medium (DMEM) supplemented with 10% fetal bovine serum (FBS), 2 mM L-glutamine, 100 U/mL penicillin, and 100 μg/mL streptomycin (all from Sigma-Aldrich) at +37 °C and 5% CO_2_. The HAPT1 AIM2 KO cell line was created using CRISPR-Cas9, as described in the Supplementary Materials and Methods.

HEK-Blue hTLR9 were acquired from Invivogen (San Diego, CA, USA). The tests were performed according to manufacturer protocols. A549 cells were cultured and infected (100 VP/cell), and the supernatant used for TLR9 activation was collected 48 h postinfection and filtered (100-kDa filter unit, Amicon Ultra 4, Merk Millipore Burlington, MA, USA) for virus removal according to protocol. Alternatively, the HEK-Blue hTLR9 cells were directly infected and measured according to manufacturers’ protocol (100 VP/mL).

Immature dendritic cells (DC) were produced from PBMCs isolated from healthy donors’ blood (Finnish Red Cross blood service, approved by the ethical board 12.03.2019, 14/2019). First, PBMCs were extracted by density centrifugation using Lymphoprep solution (Stemcell technologies, Vancouver, Canada) according to manufacturer’s instructions. Erythrocytes were removed with ACK lysis buffer (Thermo Fisher, Waltham, MA, USA). Then, cells were washed and DCs were isolated from the PBMC fraction using CD14+ magnetic beads (MACS Miltenyi Biotech, Bergisch Gladbach, Germany) according to standard procedures. Immature DCs were grown in RPMI supplemented with 10% FBS, 100 U/mL penicillin, 100 μg/mL streptomycin, 1% L-glutamate, 20 ng/mL IL-4, and GM-CSF for 6 days until usage. The CD1+ was confirmed with flow cytometry.

The design, construction, production, and purification of used viruses were described previously [6]. Briefly, all viruses are 5/3 chimeric adenoviruses with a 24-base pair deletion in the E1A region and an E2F promoter in the E1 region. To this, hIL-2, hTNFa, or hTNFa-IREs-hIL2 genes were inserted into the E3 region of the adenovirus “backbone” genome.

### 3.2. DAMP and PAMP Measurement

SK-MEL-28, OVCAR-3, or A549 cells (0.4 × 10^6^ cells/well of a 6-well plate) were infected with 100 VP/mL of unarmed, single-armed, or double-armed adenoviruses. The supernatants or the cells were collected 6, 24, or 48 h p.i. for ATP or HMGB1 measurements. ATP release was measured with ATP Determination kit Molecular probes (Invitrogen, Carlsbad, CA, USA) according to standard protocol. The HMGB1 concentration was measured by HMGB1 ELISA kit (IBL), according to manufacturer’s instructions. In turn, calreticulin cell-surface expression was measured from collected and stained cells by flow cytometry.

### 3.3. RNA-Sequencing

SK-MEL-28 cells were seeded in 6-well plates. After 24 h, OAd.TNFa-IL2 virus (100 VP/cell) and/or DCs were added to achieve a cell ratio of 1:2 (=2,000,000 DCs/well). Cells were incubated in 10% FBS, 1% Pen/Strep, and 1% L-glutamate in RPMI media, supplemented with IL4 and GMCSF for 48 h prior to sample collection. Collected cells were suspended into RNAlater (Qiagen, Valencia, CA, USA). RNA was extracted using the RNeasy Mini Kit (Qiagen, Valencia, CA, USA) according to manufacturer’s instructions. Finally, RNA quality and quantity were measured using Nanodrop 1000 (Waltham, MA, USA). The sequencing was done by BGISEQ-500RS (BGI Europe Genome Center, Amsterdam, The Netherlands).

### 3.4. NFκB Signaling Pathway Analysis

SK-MEL-28 cells were grown in T175 flasks and infected with 100 VP/cell or left uninfected (mock control). The cells were collected, and the proteins were isolated using Qproteome Mammalian Protein Prep Kit (Qiagen) according to standard protocols. Protein concentrations were determined with Bradford assay, and 50 µg of the resulting proteins was used for Proteome Profiler Human NFκB pathway Array (R&D systems, Minneapolis, MN, USA) according to protocol. The intensity of membrane proteins was determined by Image J software. 

### 3.5. Flow Cytometry

Flow cytometry preparations have been described previously [6,20]. In short, cells were first collected and washed with phosphate buffered saline (PBS). Antibodies CD1, CD3, CD11c, CD80, and CD86 (all from Biolgened, San Diego, CA, USA) were used for staining for DC maturation analysis. Staining was preformed according to manufacturer’s instructions. Calreticulin expression was measured from collected cells using Ab2907 anti-calreticulin antibody as primary antibody (IBL) and Ab 15007 Goat Anti-Rabbit IgG H&L as secondary antibody (Alexa Fluor^®^ 488) (Abcam, Cambridge, United Kingdom). Cells were analyzed using the BD Accuri C6 flow cytometer and by BD Accuri C6 software (BD biosciences, San Jose, CA, USA).

### 3.6. In Vivo Analysis

Two subcutaneous tumors (HapT1 or HapT1 AIM2 KO, 4 × 10^6^ cells/tumor) were implanted into the flanks of Syrian hamsters. The tumors were allowed to grow until reaching a size of approximately 5 mm in diameter. Then, tumors were treated by intratumoral injections every other day for a total of five times, with either PBS, unarmed oncolytic adenovirus (OAd), or OAd.TNFa-IL2, 1 × 10^9^ VPs/tumor/treatment time, diluted into a final volume of 50 µL PBS. The animals were under isoflurane anesthesia during all procedures. The tumors were collected on the 11th day of the experiment into RNAlater (Qiagen, Valencia, CA, USA) and analyzed by RTqPCR. The Experimental Animal Committee of the University of Helsinki and the Provincial Government of Southern Finland have approved the animal experiment performed in this study (Approval number ESAVI/28404/2019 date 9.10.2019).

### 3.7. Real-Time Quantitative PCR Analysis of Tumors

Tumor samples were harvested from euthanized hamsters, cut into pieces (50–100 mg/piece), and submerged in 1 mL of RNAlater (RLT buffer, Qiagen, Valencia, CA, USA) at +4 °C. RNA later was removed after 1 day, and the samples were frozen at −80 °C. Approximately 30 mg of the tissue samples was homogenized in RLT buffer, and RNA was extracted from the homogenate using the RNeasy Mini Kit (Qiagen) according to the manufacturer’s instructions. RNA (~200 ng) was converted to cDNA by reverse transcriptase (QuantiTect Rev. Transcription Kit) (Qiagen) according to manufacturer’s protocol. The produced cDNA samples were used for RTqPCR to determine the effects of AIM2 KO on the tumor microenvironment (Supplementary Materials and Methods).

## 4. Discussion

Although new and improved therapy options have been developed, cancer is still one of the leading causes of death in the world, especially, refractory tumors need further treatment options. Therefore, we need to optimize and develop methods to treat patients that are unresponsive or refractory to treatments available to date. In line with this, OAd.TNFa-IL2 was created and it has shown great promise in vivo by curing 80% of Syrian Hamsters as a single treatment and 100% of the animals when given as a combination treatment with Tumor- infiltrating lymphocyte (TIL) therapy [6]. However, the molecular mechanism behind this impressive cure rate in vivo has not been fully determined. Therefore, this study set out to dissect the mechanism behind this observed effect in order to further enhance the therapy and to determine the biology behind the reaction. Moreover, understanding the mechanisms of this virus might help in selecting responsive patient populations for the treatment.

To investigate this matter, we determined whether OAd.TNFa-IL2 could induce PRR-activating DAMP- and PAMP-molecule release. Our study shows that OAd.TNFa-IL2 induced both extracellular ATP and HMGB1 release and increased cell surface expression of calreticulin. Normally, the cytosol-located molecular chaperon calreticulin binds to and targets proteins for destruction, but when secreted, it has been shown to promote lymphocyte infiltration and thus mediate antitumoral effects [21]. Additionally, extracellular ATP and HGBM1 have been linked to immune cell activation [22]. Therefore, we can conclude that one mechanism behind the noted antitumor efficacy of OAd.TNFa-IL2 seems to be DAMP- and PAMP-signaling mediated.

It has been shown that chronic high-level expression of alarmins can be associated with carcinogenesis [23]. In contrast, dynamic, acute, and regulated expression is conducive to immune response. These phenomena are relatively well understood for example for ATP [24,25]. Therefore, it is not expected that immunologic signaling molecules such as alarmins would be highly expressed all the time in response to immunostimulatory therapy such as oncolytic virus. In light of this information, three time points for SK-MEL-28 alarmin release were investigated. The results show a trend for but not significantly higher expression of alarmins. As each time point represents a snapshot, lack of significant alarmin increase is not indicative of lack of the phenomenon at some other time point. Thus, the measured time points might have just missed the peak release time point of this cell line.

Since alarmins can activate a plethora of signaling cascades, we further examined the response by RNA sequencing to investigate which signaling pathways and PRRs were activated by virotherapy. We confirmed that the virus did activate several DAMP- and PAMP-signaling pathways; however one PRR, an inflammasome called AIM2, was activated in both DC and SK-MEL28 cells (unfiltered results) and was thus investigated further.

It has been shown in an in vivo mouse model that similar adenoviruses, but armed with CpG island, induce antitumor efficacy through the TLR9 [26]. Therefore, the effect of OAd.TNFa-IL2 on TLR9 activation was further analyzed by HEK-blue.hTLR9 cell assay and NFκB analysis. Transient DC TLR9 activation has been associated with positive outcome; however, TLR9 signaling in, e.g., T cell or cancer cells can lead to tumor growth enhancement [27]. This study revealed that the NFκB pathway was generally downregulated by OAd.TNFa-IL2infection and that TLR9 was not activated by the virus. Thus, these data indicate that TLR9 may not be the primary sensor of OAd.TNFa-IL2 infection and implicates that other pathways are responsible for the noted favorable tumor microenvironment modulation during therapy. Interestingly, the supernatant of OAd.TNFa-infected cells induced TLR9 receptor activation. One might speculate that virus-encoded TNFa might cause immunogenic cell death, which is then seen as TLR9 activation in the supernatant group, but when challenged by the virus alone, the effect had not enough time to develop. Additionally, the NFκB-signaling pathway analysis revealed that the ASC protein in AIM2 inflammasome was upregulated, further validating the RNA-sequencing results.

From these analyses, AIM2 rose as a lead PRR candidate for danger-signaling mediator during OAd.TNFa-IL2 infection. It has been shown that AIM2 senses cytosolic dsDNA, and thus, the dsDNA genome of OAd.TNFa-IL2 is the likely activator of AIM2. Binding of dsDNA to the AIM2 HIN200 domain leads to its multimerization through a linked PYD domain and to the formation of a helical-like structure. This multimeric structure recruits and activates caspase-1, which splices pro-IL-1beta and pro-gasdermin into their active forms. Gasdermin forms pores in cell membranes that can lead to cell death by pyroptosis. Simultaneously, pore formation facilitates IL-1beta secretion into the extracellular space, creating a pro-inflammatory environment.

When analyzed in vivo, AIM2 KO had major effects on cytokine profiles in tumors. Marked difference in cytokine expression levels between AIM2 KO and wild type tumors was seen in IL-1, a downstream AIM2 cytokine. In “wt“ HapT1 tumors, IL1beta expression was high, while the opposite was true for KO tumors. IL-1 has been claimed to be a T cell proliferation-inducing, co-stimulatory cytokine, consequently triggering IL-2 secretion and expression of IL-2Rs by activated T cells. These previous notions are in line with the results of our study. IL-1beta levels were significantly upregulated in OAd.TNFa-IL2-treated tumors, and the same trend could be noted for IL-2 and its receptor. Subsequently, this indicates that AIM2 activation plays an important role in virotherapy-induced growth inhibition by secretion of inflammatory cytokines, which attract and activate immune cells against tumor cells.

Additionally, OAd.TNFa-IL2 may induce antitumor effects through trough AIM2 activated, gasdermin-induced cell killing, with associated immunogenic tumor microenvironment modulation. AIM2 knockout-specific reactions in cytokine measurements could also be seen in IL-6 levels; IL-6 expression was abrogated in KO tumors and shows an interesting link between AIM2 and IL-6 expression. Previous studies on IL-6 reveal to be a multifaceted player in cancer; it can recruit neutrophils and T cells, but it can also activate several cancer cell growth-inducing signaling pathways [28]. Furthermore, IL-6 can affect IL-2 signaling and thus inhibit T cell recruitment and activation [29].

In virotherapy-treated tumors, a trend of higher IL-2 expression could be noted. IL-2 plays an essential role in the immune system, affecting tolerance and immunity, primarily via its effects on T cells [30]. IL-2 promotes the differentiation of T cells into effector and into memory T cells after antigen stimulation and, consequently, was chosen as one of the two cytokines added into the OAd-arming device. The other part of the OAd.TNFa-IL2-arming device, TNFa, is a cytokine that is involved in both local and systemic inflammation. It is produced chiefly by activated macrophages, although it can be produced by many other cell types as well. It has a plethora of effects; however, it is well known for its cell destroying properties [31]. Thus, the high expression of these cytokines in Syrian hamsters during OAd.TNFa-IL2 infection in vivo likely promoted the noted tumor growth limitation.

Further analysis of tumors revealed an additional plausible mechanism of how OAd.TNFa-IL2 contributes to antitumor efficacy. OAd.TNFa-IL2 induced expression of CD83 in tumors. CD83 is an established DC maturation marker. DC maturation leads to enhanced antigen presentation, which plays an important role in mediating antitumor reactions by T cells.

In summary, this paper shows that OAd.TNFa-IL2 adenovirus virotherapy affects the tumor microenvironment through DAMP and PAMP release and subsequent activation of sensors, such as AIM2. These effects may underlie the efficacy of OAd.TNFa-IL2 and could be relevant for many other oncolytic viruses as well.

## Figures and Tables

**Table 1:** In vitro assessment of intracellular virus-induced signaling in OAd.TNFa-IL2-infected SK-MEL-28 melanoma cells or dendritic cells (DCs), evaluated by RNA-sequencing: Top 8 genes showing the most marked upregulation in RNA expression levels as measured through RNA-sequencing (Poisson distribution filtering applied) are listed. Note that the virus codes for TNFa and IL-2 but transgene expression is linked to replication, which only occurs in tumor cells.

SK-MEL-28				
Gene	Abbreviation	Log2 Ratio (Infected versus Uninfected Cancer Cells)	Up- or Downregulation	*p*-Value
Interleukin 2	IL-2	11.620	Up	0
Tumor necrosis factor alfa	TNFa	11.388	Up	0
Ubiquitin	UBD	9.958	Up	1.284006 × 10^−182^
Histone H2A type 1	HIST1H2AI	9.935	Up	1.307986 × 10^−08^
Chemokine (C-C motif) ligand 2	CCL2	9.527	Up	6.97892 × 10 ^−71^
Abhydrolase domain containing 14A and aminoacylase 1 (read-through transcription)	ABHD14A-ACY1	8.991	Up	1.422174 × 10^18^
Myotrophin	MTPN	8.969	Up	1.681326 × 10^−38^
Interleukin 32	IL32	8.394	Up	9.93188 × 10^−74^
**DC**				
**Gene**	**Abbreviation**	**Log2 Ratio (Infected versus** **Uninfected DC)**	**Up or Down-Regulation**	***p*** **-Value**
Myothrophin	MTPN	9.629	Up	3.8971 × 10^−65^
Locus101929802	LOC101929802	6.931	Up	9.91084 × 10^−14^
Locus102724994	LOC102724994	6.919	Up	8.93212 × 10^−07^
Zink finger816-Zink finger321P	ZNF816-ZNF321P	6.443	Up	1.447082 × 10^−05^
Heat shock 10kDa protein 1 and MOB family member 4, phocein (read-through transcription)	HSPE1-MOB4	5.807	Up	1.447082 × 10^−05^
Nuclear Pore Complex Interacting Protein Family Member A3	NPIPA3	4.518	Up	1.870752 × 10^−16^
Chemokine (C-X-C motif) ligand 9	CXCL9	3.0	Up	2.73386 × 10^−10^
Platelet glycoprotein Ib alpha chain	GP1BA	2.269	Up	7.3774 × 10^−106^

## Supplementary Materials

The following are available online at https://www.mdpi.com/2073-4409/9/4/798/s1, Figure S1: DAMP AND PAMP expression in OAd (with or without arming device) infected cells (100VP/cell) was measured on three time points (6-, 24 and 48 h p.i.), Figure S2: Dendritic cell maturation through agonist stimulation. Cells were incubated for 24h with or without agonist or antagonist or virus before staining for DC maturation markers A) CD86 and B) CD80 and measurement by flow cytometry, Figure S3: Sequencing of HapT1 CRISPR/Cas9 KO of AIM2, compared to unedited HapT1 cell line, Figure S4: Virus induced matured IL-1beta release. IL-1beta release into infected cell supernatants, Table S1: Gene expression of receptors and proteins involved in DAMP- and PAMP –signaling, measured by RNA-seq.
