# Supplementary material for: TNFa and IL2 Encoding Oncolytic Adenovirus Activates Pathogen and Danger-Associated Immunological Signaling
Source: Cells. 2020 Mar 26;9(4):798. doi: 10.3390/cells9040798 (PMC7225950; doi:10.3390/cells9040798)
Supplement: Supplementary file 1 [file cells-09-00798-s001.pdf]

Supplementary Materials and methods

RTqPCR

For cytokines and maturations markers, all qPCR probes and oligos (forward and reverse) were purchased from Metabion (Table x) and designed by Zivcec *et al.*[29]. PCR reactions were carried out using LightCycler® 480 Probes Master, Roche, at a denaturation temperature of 95 °C for 30 sec, annealing temperature of 50 °C for 30 sec, and an extension temperature of 72 °C with an extension time of 30 sec for 40 cycles.

AIM2 antagonist and agonist effects on dendritic cells

SK-MEL-28 cells (100 000 cells/well), were seeded on 6-well plates. After 24h, immature DCs were added (1:1 ratio), with or without antagonist Ac-YVAD-cmk (10 μg/ml) or Poly Lyo Vec-complex (1 μg Poly dA:dT per 100 μl of LyoVec, 50 μl of complex/ml) (both from InvivoGen) for 24 h and analyzed by flow cytometry (BD Accuri). LPS (100 ng/ml, from Sigma) was used as a positive control.

CRISPR/Cas9 KO cell line

HAPT1 AIM2 KO cell line was created as follows: Ribonucleoprotein complexes (RNPs) consisting of Alt-R® CRISPR-Cas9 tracrRNA, Alt-R® CRISPR-Cas9 crRNA (spacer sequence GAACAACUGAUUGAGAGUGC) and Alt-R® S.p. HiFi Cas9 Nuclease V3 (Integrated DNA Technologies, IA) were prepared according to the manufacturer's protocol. The RNPs were delivered into HAPT1 cells using the Neon Transfection System 10 μL Kit with the Neon Electroporation Transfection system (ThermoFisher, MA), settings 1200 Volt/20 milliseconds/2 pulses. One day after RNP delivery, cells were seeded in 96-well plates (30 cells/plate), and derived clonal populations were assayed for successful editing of the target site (GAACAACTGATTGAGAGTGCAGG, 150 – 172 bp downstream of the AIM2 start codon), by PCR-amplifying the region between 256 bp upstream and 252 bp downstream of the target site in the genomic DNA (primers AAGACAATGGGTGGCAGTCC and TTCTGTGGAGCACTCACCTT, Kapa HiFi HotStart 2x Readymix PCR kit, Roche, Switzerland) and Sanger-sequencing (primer GCGGAGGAATTTCTAACACTCCTTTTA, Eurofins Mix2Seq kit, Eurofins, Luxembourg). The selected clone was found to have a homozygous 5 bp deletion directly upstream of the PAM-sequence of the target site leading to out-of-frame deletion mutation GAACAACTGATTGAG-----AGG.

Release of mature IL-1beta

SK-MEL-28 and A549 cells were infected with OAd.TNFa-IL-2 (1000VP/cell) and cell culture supernatants were analysed by ELISA (Invitrogen IL1Beta kit) 24 h.p.i. indicating active, plausibly AIM2 dependent, cleavage and release of IL-1beta.

Supplementary results

DAMP and PAMP molecule secretion and expression

Several alarmins were secreted or expressed during OAd.TNFa-IL2 infection (Supplementray Figure 1)

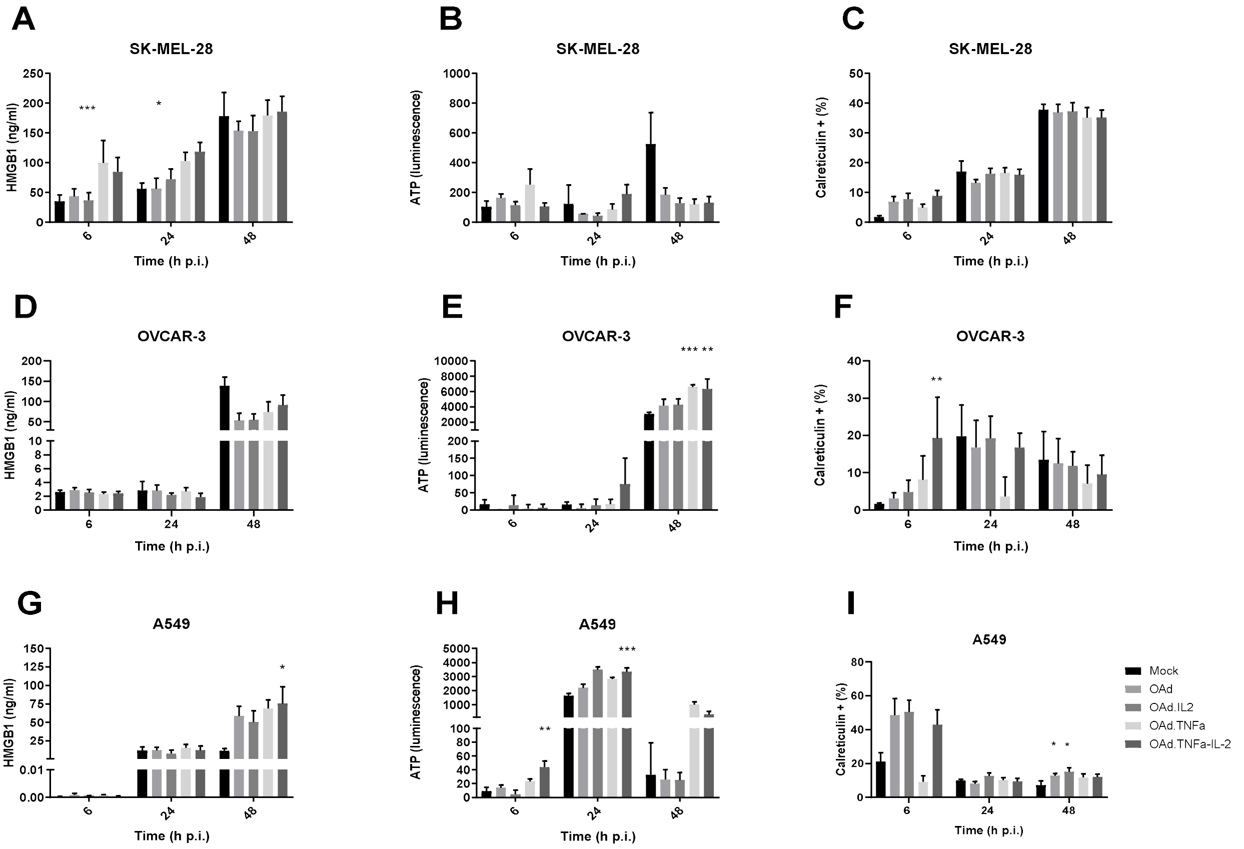

Supplementary Figure 1. DAMP AND PAMP expression in OAd (with or without arming device) infected cells (100VP/cell) was measured on three time points (6-, 24 and 48 h p.i.). in three cell lines; (A-C) SK-MEL-28 cells, (D-F) OVCAR-3 cells, and (G-F) A549 cells. ATP and HMGB1 release was analyzed by ELISA, while Calreticulin expression was analyzed by flow cytometry. Kruskall-Wallis, SEM,*p<0.05, **p<0.01, ***p<0.001, ****p<0.0001.

OAd.TNFa-IL2 induces DAMP- and PAMP-signaling cascade related protein gene expression in both SK-MEL-28 and dendritic cells

Several genes encoding for DAMP- and PAMP- realted pathways were upregulated during virotherapy when analyzed *in vitro* (Supplementary Table1).

Supplementary Table 1 Gene expression of receptors and proteins involved in DAMP- and PAMP –signaling, measured by RNA-seq.

SKMEL-28 VS INFECTED SKMEL-28		
NON-FILTERED	GENE	FILTERED
Up	ADORA1	0
Up	ADORA2A-AS1	0
Down	ADORA2B	0
0	ADORA3	0
Down	AGER	0
Up	AIM2	Up
0	CD91	0
0	cGAS	0
Up	DDX41	0
0	FPR1	0
Up	FPR2	0
Down	FPR3	Down
Down	HAVCR2 (TIM-3)	0
Up	IRF1	UP
Down	IRF2	0
Up	IRF3	0
Down	IRF4	0
Up	IRF5	0
Down	IRF6	0
Up	IRF7	0
0	MyD88	0

DC VS infected DC		
NON-FILTERED	GENE	FILTERED
Up	ADORA1	0
Up	ADORA2A	0
Up	ADORA2A-AS1	0
Down	ADORA2B	0
Down	ADORA3	Down
Up	AIM2	0
Up	CD40	0
Up	CD40LG	0
Up	CD80	0
Up	CD83	0
Up	CD86	0
0	cGAS	0
Down	DDX41	0
Down	FPR1	0
Up	FPR2	0
Down	FPR3	0
Down	HAVCR2 (TIM-3)	0
Up	IRF1	0
Up	IRF2	0
Down	IRF3	0
Up	IRF4	0

Up	NLRP1	0
Up	NLRP2	0
Down	NLRP3	0
Up	NOD2	0
Down	P2RX7	0
Down	P2RY2	0
Up	QRFPR	0
0	RIG-I	0
Down	STING (TMEM173)	0
Up	TLR1	0
Up	TLR2	0
Up	TLR3	0
0	TLR4	0
Up	TLR5	0
Down	TLR6	0
Up	TLR7	0
0	TLR8	0
Up	TLR9	0
Up	TLR10	0

Down	IRF5	0
Up	IRF6	0
0	IRF7	0
Down	MYD88	0
Down	NLRP1	0
Down	NLRP2	0
Down	NLRP3	0
Down	P2RX7	0
Up	P2RY2	0
Down	QRFPR	0
0	RIG-I	0
0	STING (TMEM173)	0
Down	TLR1	0
0	TLR10	0
Up	TLR2	0
Down	TLR3	0
Down	TLR4	0
Down	TLR5	0
Down	TLR6	0
Up	TLR7	0
Down	TLR8	0
	TLR9	0

OAd.TNFa-IL2 causes immune cell activation through AIM2 signaling

In order to determine the effect of OAd.TNFa-IL2 -induced AIM2 signaling on DC maturation, we analyzed immature DCs exposed to OAd.TNFa-IL2 , co-cultured with or without SK-MEL-28 melanoma cells and with or without AIM2 downstream antagonists (Ac-YVAD-cmk) or agonist (complex of LyoVec/PolydA:dT). On average, 97.2% of the immature DCs used for this study were CD1+. The results showed that stimulation of the signaling cascade with an agonist lead to an increase in CD80+ cells (C, DC, AD+ group MFI mean= 64335, mock=27995, not significant) and CD86+ cells (C, DC, AD+ group mean MFI= 114400, compared to mock mean MFI =19366, not significant) highlighting the importance of this signaling cascade in activation of DCs.

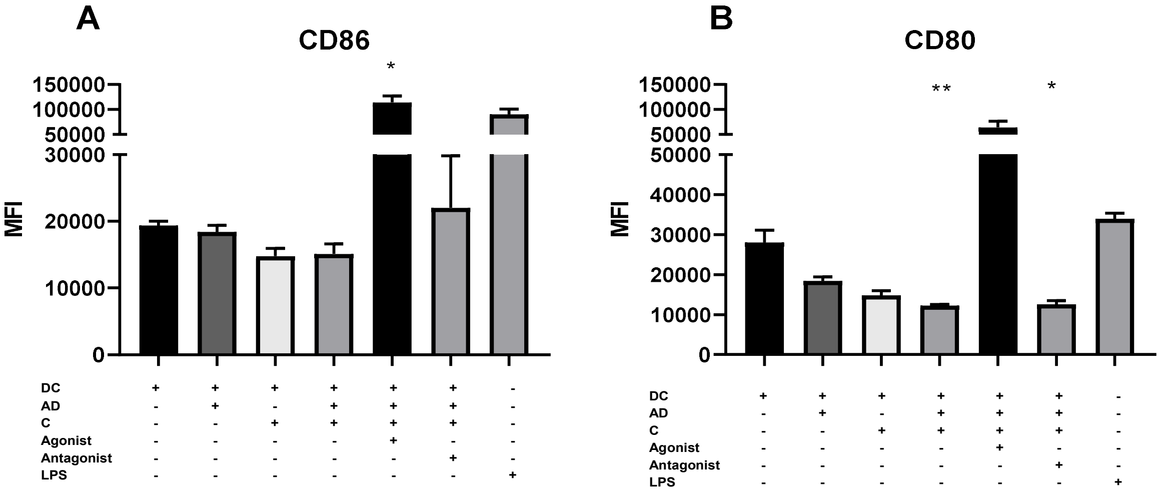

Supplementary Figure 2. Dendritic cell maturation through agonist stimulation. Cells were incubated for 24h with or without agonist or antagonist or virus before staining for DC maturation markers A) CD86 and B) CD80 and measurement by flow cytometry. DC= dendritic cells, C= cancer cells (SK-MEL-28), AD= Adenovirus (OAd.TNFa-IL2), LPS= Lipopolysaccharide, Agonist = LyoVec/Poly(dA:dT) complex, Antagonist = Ac-YVAD-cmk). Data are presented as mean + SEM, n > 6. Statistical analyses were done by Kruskal-Wallis test, treatments compared to mock, *p=0.05, **p=0.01.

Verification of AIM2 KO

CRISPR/CAS9 modified HapT1 clone was sequenced in order to confirm successful KO of AIM2 gene (Supplementary Figure 5), and a deletion was confirmed.

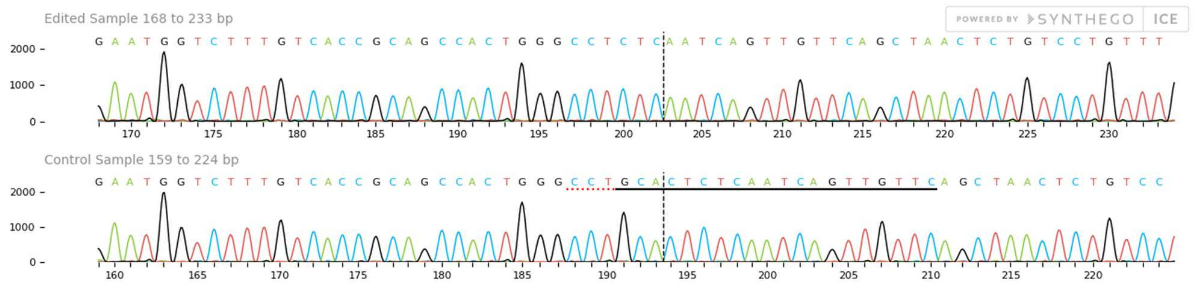

Supplementary Figure 3. Sequencing of HapT1 CRISPR/Cas9 KO of AIM2, compared to unedited HapT1 cell line.

Activation of AIM2

To verify that virotherapy does not only activate AIM2 protein production, but can induce the formation of active inflammasomes, the concentration of matured and secreted IL-1beta, the endproduct of AIM2 activation, was measured. The average concentration of IL-1beta was 1,3 and 1,9 times higher in infected SK-MEL-28 and A549 cell supernatants respectively (p=0,3 and 0,001), compared with mock infected cells.

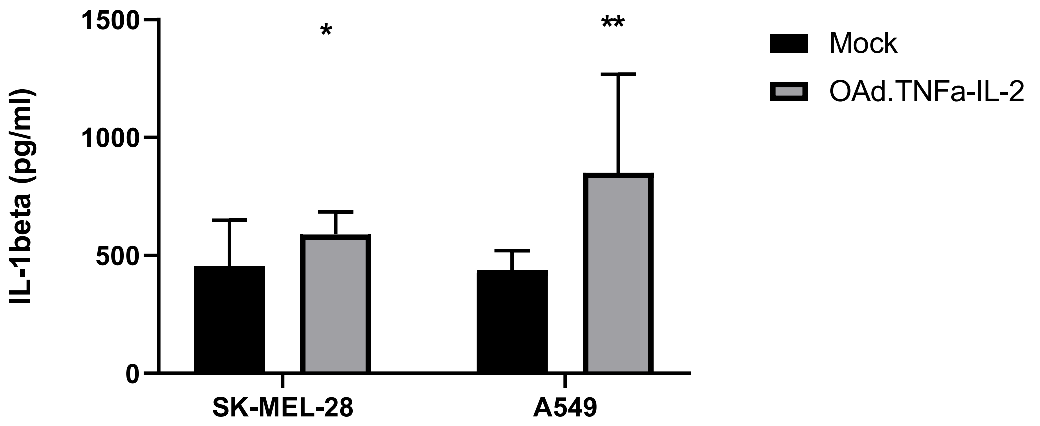

Supplementary Figure 4. Virus induced matured IL-1beta release. IL-1beta release into infected cell supernatants. Cells were infected with OAd.TNFa-IL-2 (1000 VP/cell) and cell culture supernatants were analysed by ELISA, indicating plausibly AIM2 dependent, cleavage, activation and release of IL-1beta. Data are presented as mean + SEM, Kruskal-Wallis test, treatments compared to mock of same cell line, *p=0.05, **p=0.01.
